# MRI-guided, transrectal, intraprostatic steam application as potential focal therapeutic modality for prostatic diseases in a large animal translational model: A feasibility follow-up study

**DOI:** 10.1371/journal.pone.0226764

**Published:** 2019-12-23

**Authors:** Adriano Wang-Leandro, Florian Willmitzer, Agnieszka Karol, Beat Porcellini, Peter Kronen, Emile M. Hiltbrand, Daniel Rüfenacht, Patrick R. Kircher, Henning Richter

**Affiliations:** 1 Clinic for Diagnostic Imaging, Department for Clinical Diagnostics and Services, Vetsuisse Faculty, University of Zurich, Zurich, Switzerland; 2 Vetzentrum AG, Zurich, Switzerland; 3 Department of Molecular Mechanisms of Disease, Vetsuisse Faculty, University of Zurich, Zurich, Switzerland; 4 Radiology Department, Hirslanden Clinic, Zurich, Switzerland; 5 Veterinary Anaesthesia Services-International (VAS-int.), Winterthur, Switzerland; 6 Cermavein, Archamps, France; National Institutes of Health, UNITED STATES

## Abstract

Parallel to establishment of diagnostic surveillance protocols for detection of prostatic diseases, novel treatment strategies should be developed. The aim of the present study is to evaluate the feasibility and possible side effects of transrectal, MRI-targeted intraprostatic steam application in dogs as an established large animal translational model for prostatic diseases in humans. Twelve healthy experimental, intact, male beagle dogs without evidence of prostatic pathology were recruited. An initial MRI examination was performed, and MRI-targeted steam was applied intraprostatically immediately thereafter. Serum levels of C-reactive protein (CRP), clinical and ultrasonographic examinations were performed periodically following the procedure to assess treatment effect. Four weeks after treatment, all dogs underwent follow-up MRI examinations and three needle-core biopsies were obtained from each prostatic lobe. Descriptive statistics were performed. MRI-guided intraprostatic steam application was successfully performed in the study population. The first day after steam application, 7/12 dogs had minimal signs of discomfort (grade 1/24 evaluated with the short-form Glasgow Composite Measure Pain Scale) and no dogs showed any sign of discomfort by day 6. CRP elevations were detected in 9/12 dogs during the first week post steam application. Mild to moderate T2 hyperintense intraparenchymal lesions were identified during follow-up MRI in 11/12 dogs four weeks post procedure. Ten of these lesions enhanced mild to moderately after contrast administration. Coagulative necrosis or associated chronic inflammatory response was detected in 80.6% (58/72) of the samples obtained. MRI-targeted intraprostatic steam application is a feasible technique and displays minimal side effects in healthy dogs as translational model for human prostatic diseases. This opens the possibility of minimally invasive novel treatment strategies for intraprostatic lesions.

## Introduction

Prostate cancer (PCa), the most common non-cutaneal neoplasia diagnosed in men and is a leading cause of death worldwide representing significant social and economic burdens [1–3].

Screening with Prostate Specific Antigen (PSA) has led to an increasing number of patients diagnosed with small volume, low grade cancer [4]. Patients and clinicians are faced with the dilemma how to treat localized prostate cancer. Traditionally, prostate cancer has been treated with radical surgery or irradiation, each providing excellent long-term tumor control, but they are accompanied by the risk of incontinence and impotence [5]. Organ sparing therapies are now common for breast, skin and kidney tumors, resulting in equivalent cancer control but lower morbidity rates and better quality of life [6–8]. Focal cancer therapy strongly depends on imaging modalities which confidentially identify the cancer lesion, evaluate the results and monitor for treatment failure. Improvements in multiparametric magnetic resonance imaging (MRI) have improved the ability to detect clinically significant cancer over the past decade [9–12].

In parallel, focal prostatic thermal ablation has become a research focus, as it represents a minimally invasive treatment possibility for PCa. The goal of thermal-dependent procedures is to generate targeted coagulative necrosis to the neoplastic prostatic tissue while sparing integrity and function of non-affected tissue, urethra and periprostatic structures [13]. Intraprostatic focal application of water vapor generates convective heat distribution within the treated area, specifically being distributed extracellularly and transferring heat onto the cellular membranes [14]. Magnetic resonance imaging (MRI) represents a versatile and highly sensitive tool for detailed evaluation of the prostatic parenchyma and follow-up examinations after prostatic lobe ablation [15, 16]. Additionally, MRI facilitates efficient monitoring of targeted diagnostic or therapeutic approaches of the prostate gland [16–22].

Over the last years, the role of the dog as a large animal translational model for human benign prostatic hyperplasia (BPH) and PCa has gained recognition to a greater extend due to anatomical, clinical, histopathological and epidemiological similarities between these two species [23–25].

The aims of the present prospective-designed study are: (1) to evaluate the feasibility of transrectal, MRI-guided, intraprostatic steam application as a potential technique for focal treatment of prostatic disease in a population of healthy dogs as a large animal translational model, (2) to clinically assess and report any possible side effects related to the technique in a follow-up period of 4 weeks, and (3) to describe the effects of steam application within prostatic parenchyma using histopathology after a follow-up prostatic biopsy. We hypothesise that intraprostatic steam application is a feasible technique in dogs, showing minimal-to-none clinically evident side effects. We further hypothesise that this technique will enable a focal intralobar coagulative necrosis.

## Materials and methods

### Animals

Twelve healthy, intact male experimental beagle dogs hosted by the Vetsuisse Faculty of the University of Zürich were selected for the study. Dogs had a mean weight of 14.98 kg (range: 13–18.4 kg) and a mean age of 5.5 years (range: 2.2–8 years).

Inclusion criteria had to be considered as normal and included a physical examination, complete blood count (CBC), serum biochemistry analysis, c-reactive protein (CRP) values and ultrasound examination of the prostate gland. The study was conducted in accordance to the guidelines of the Animal Welfare Act of Switzerland and approved by the Cantonal Veterinary Office of Zurich (permission number: ZH026/16).

### Anaesthesia

MRI-guided intraprostatic steam application was performed under general anaesthesia. The anaesthetic protocol included a premedication with butorphanol (0.2mg/kg) and medetomidin (0.02 mg/kg) i.m. Approximately 10 minutes thereafter, a venous catheter was placed in the cephalic vein and anaesthesia was induced with a combination of midazolam (0.1 mg/kg), lidocaine (1–3 mg/kg), ketamine (1–2 mg/kg), and propofol (1–6 mg/kg) intravenously. An endotracheal tube was placed, and maintenance of the anaesthesia was achieved using sevoflurane (3–5% FiO_2_) and a constant rate infusion of propofol (0.01–0.06 mg/kg/min).

Anaesthesia was performed by a board-certified veterinary anaesthetist and systemic parameters (heart rate, respiratory rate, blood pressure, oxygen saturation and body temperature) were monitored during the entire duration of the procedure.

### Image acquisition and evaluation

Ultrasound examinations of the prostate gland were performed using a 10 (5–13) MHz wideband linear probe (GE LOGICe ultrasound machine, GE Healthcare, Glattbrug, Switzerland). Echogenicity, echoarchitecture, and contour of the prostate were evaluated and documented as still images.

**Fig 1:**
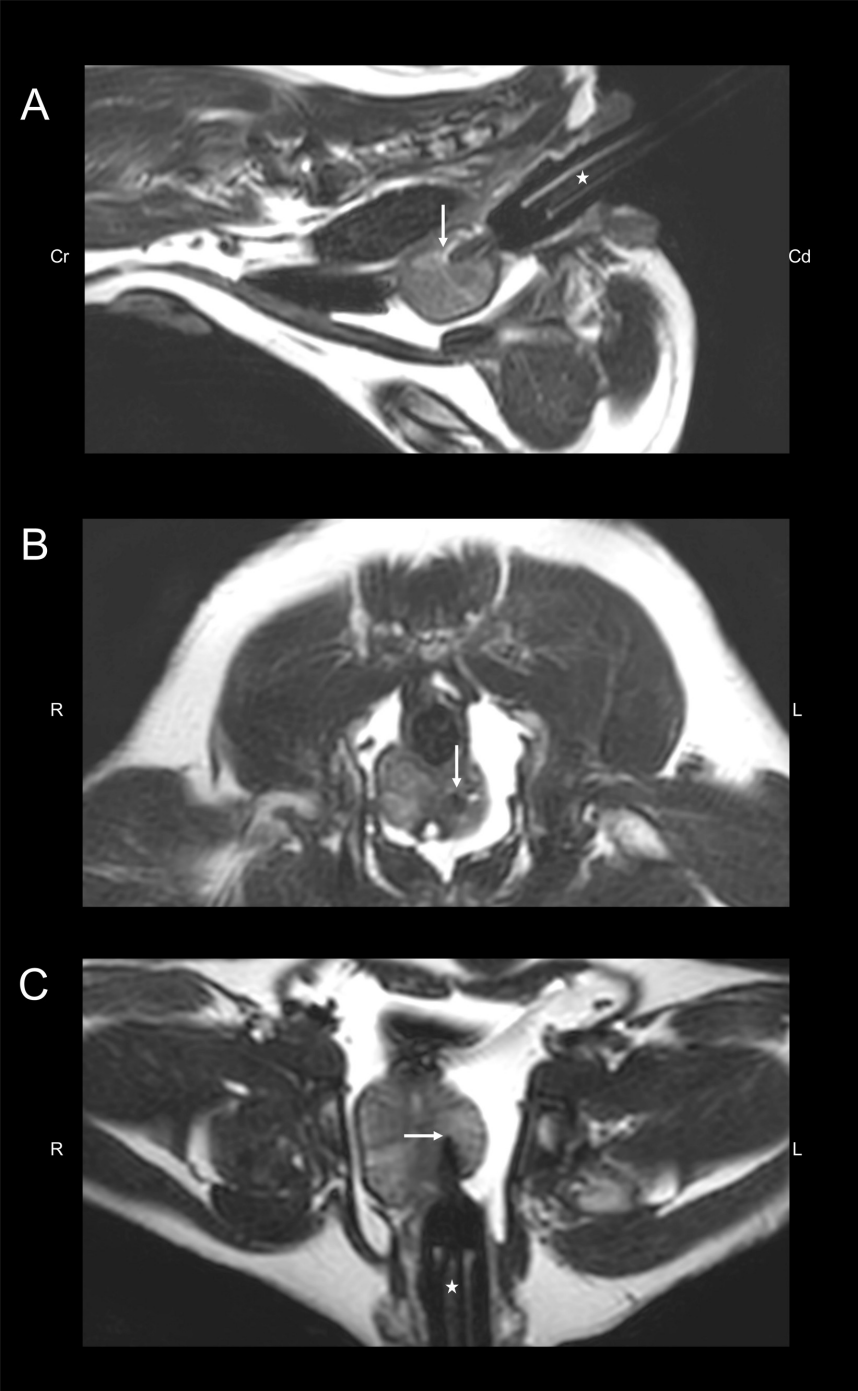
Sagittal, dorsal and transverse verification T2-weighted sequences of a 6-year-old healthy beagle during transrectal intraprostatic steam application. The white star indicates the position of the gadolinium-marked tube. The white arrow points at the tip of the needle adapted to the tube and therefore the treatment focus. Abbreviations: Cd, caudal; Cr, cranial; L, left, R, right.

MRI sequences performed included T2-weighted (T2W) in sagittal (TR 2971 ms; TE 110 ms), dorsal (TR 2433 ms; TE 110 ms), and transversal planes (TR 2438 ms; TE 110 ms), 3D T1-weighted (T1W; TR 5.35 ms; TE 2.75 ms) and post-contrast 3D-T1W spectral attenuated inversion recovery (SPAIR; TR 5.35 ms; TE 2.75 ms). Gadolinium (Omniscan, GE Healthcare AG, Opfikon, Switzerland) was applied intravenously as contrast agent (0.1 mmol/kg). Fat suppressed 3D-T1W SPAIR allows better evaluation of contrast medium distribution, specially near the periphery of the prostate gland, as identification of increased contrast uptake may be challenging due to presence of periprostatic fat. For targeted placement of the steam catheter and biopsy needle, verification T2W images were additionally acquired in the three planes using a fast image acquisition (Fig 1).

MRI evaluation of the prostate gland pre- and 4 weeks post-intervention included: signal intensity in T2W sequences, contrast uptake in 3D SPAIR-T1W sequences, presence and distribution of lesions and signal intensity changes in the surrounding tissues. The medial iliac lymph nodes were assessed for post-intervention lymphadenomegaly; for this, the maximal width and height were measured in transverse post contrast SPAIR-T1W images and compared to pre-interventional measurements.

All images were stored as DICOM files and reviewed using a free-available software (Horos Project, version 2.2.0).

## Methods

All recruited dogs of the study population met the inclusion criteria. At inclusion, dogs were randomized and divided into two different groups. The first group (n = 6) received a centrally targeted intraprostatic steam application in each prostate gland lobe, whereas the second group (n = 6) received a peripherally targeted application.

Intraprostatic steam application was performed by means of an MRI guided transrectal approach using a customized 17-gauge titanium needle and a customized 18-gauge PEEK catheter with four micro holes located 1mm from its tip. For this, dogs were positioned in sternal recumbency with the pelvic region ventrally supported and slightly elevated. MRI examination of the prostate gland was performed with a 3-Tesla scanner using 8 channel flex coils (scanner: Philips Ingenia, coils: dStream Flex M Coil; Philips AG, Zurich, Switzerland). To perform transrectal targeted positioning of the PEEK catheter, the DynaTRIM® interventional device (Invivo, Schwerin, Germany) was implemented. The PEEK catheter was placed inside a titanium needle adapted to a gadolinium-marked plastic tube and subsequently directed intrarectally to the prostate gland; furthermore, position of the needle was MRI-verified before steam application by means of T2-weighted sequences (Fig 1).

Prostatic steam ablation was performed using a VenoSteam System® (CermaVein S.A.S., Archamps, France; U.S patent number 9011367). In both groups, 10 steam pulses were applied within the right prostatic lobe and 15 within the left prostatic lobe. Moreover, sterile water was used, and each applied pulse was standardized at 80μl of water vapour at 150°C.

Dogs received antibiotic therapy over 7 days starting the day of the intervention (Amoxicilin/clavulanic acid; 12.5mg/kg BID) and were daily evaluated for any sign that could be interpreted as pain or discomfort using the short-form Glasgow Composite Measure Pain Scale (CMPS-SF) during a minimum period of 22 days [26]. CMPS-SF ranges from 0 to 24, the latest representing the maximum level of pain (CMPS-SF as described by Reid and colleagues available at http://www.newmetrica.com/wp-content/uploads/2016/09/Reid-et-al-2007.pdf). A cut-off value of 5 points was set for implementation of further analgesic modalities. Moreover, values of CRP were measured daily during the first week post ablation and at days 12, 19, and 28 post intervention.

**Fig 2:**
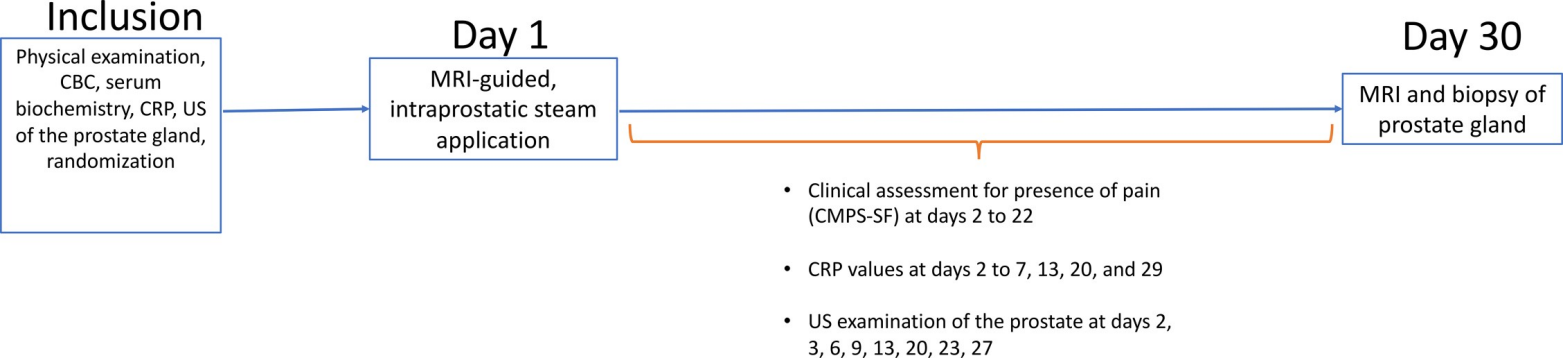
Study design. Abbreviations: CBC: complete blood count; CRP: c-reactive protein; US: ultrasound; MRI: magnetic resonance imaging; CMPS-SF: short form of the Glasgow composite measure pain scale.

In order to assess the long-term effect of intraprostatic steam, dogs underwent a second MRI examination of the prostate gland application four weeks after prostatic ablation (Fig 2), including the same sequence protocols. Moreover, using the same interventional device, targeted prostatic biopsies were obtained MRI-guided via transrectal puncture using a titanium Fully Automatic Biopsy Gun (3T MRI 150/18, Invivo, Schwerin, Germany). Three biopsy samples were collected from the lesioned area within each prostatic lobe.

### Histopathological evaluation

Prostate gland tissue obtained during the biopsy procedure was placed within embedding plastic cassettes and fixed by immersion in 4% neutral buffered formalin. Afterwards, tissue was embedded in paraffin and sectioned at 3–5 μm thick slices. Fixed tissue underwent Hematoxilin and Eosin staining and was evaluated using light microscopy. Collected biopsies were evaluated for the quality of the material obtained. Quality of the samples were classified as low (20% can be evaluated), moderate (20–50% can be evaluated), good (51–70% can be evaluated) very good (71–99% can be evaluated), or excellent (100% all can be evaluated). Furthermore, histopathological description of the tissue included tissue architecture (cell types and morphology), presence of inflammation and classification if present (for temporality: acute, subacute, chronic, chronic-active; for severity: mild, moderate or severe; for distribution: localised, multifocal, diffuse), presence of necrosis and classification if present (type: coagulative or liquefactive; for distribution: localized, multifocal, diffuse) and presence of fibrosis or scaring. If present within the collected sample, transition zone (TZ) between lesioned and apparently unaffected prostatic tissue was classified as sharp or smooth.

Long-term follow-up (eleven months after intraprostatic steam application) histopathologic examination of the whole prostate gland was available in one dog, as it was euthanized as a part of another independent study. The euthanasia was performed according to the swiss animal welfare act. At the end of this experiment the dog was euthanized by injection of pentobarbital (0.5 ml/kg) intravenously, while still under anaesthesia.

### Statistical analysis

Descriptive statistics were performed for population characteristics. Non-parametric variance analysis to compare the size of the lymph nodes in the pre and post-interventional time points were performed using Mann-Whitney tests. Statistical analysis of data was performed using commercially available software (IBM SPSS Statistics for Windows, version 21.0, IBM Corp, Armonk, NY, USA).

## Results

### Clinical evaluation

Pain assessment using CMPS-SF revealed that 7/12 dogs had minimal signs of discomfort (grade 1/24) during the first day after steam application, which were evidenced by a decreased level of activity and/or intermittently looking towards the base of the tail. The number of dogs still showing grade 1 decreased to 4 and 3 during the second- and third-day post intervention, respectively. Two dogs remained showing signs compatible with CMPS-SF grade 1 until the fifth day posterior to the prostatic ablation. None of the dogs had any detectable sign of pain (grade 0/24) after the fifth day post intervention. Additional analgesic treatment was not needed in any case. From the 7 dogs presenting any sign of pain, 5 dogs belonged to group 1. Moreover, reduction in the food intake was noticed in 2 dogs during the first and second day after prostatic ablation. No signs of urine incontinence, haematuria or stranguria were observed.

**Fig 3:**
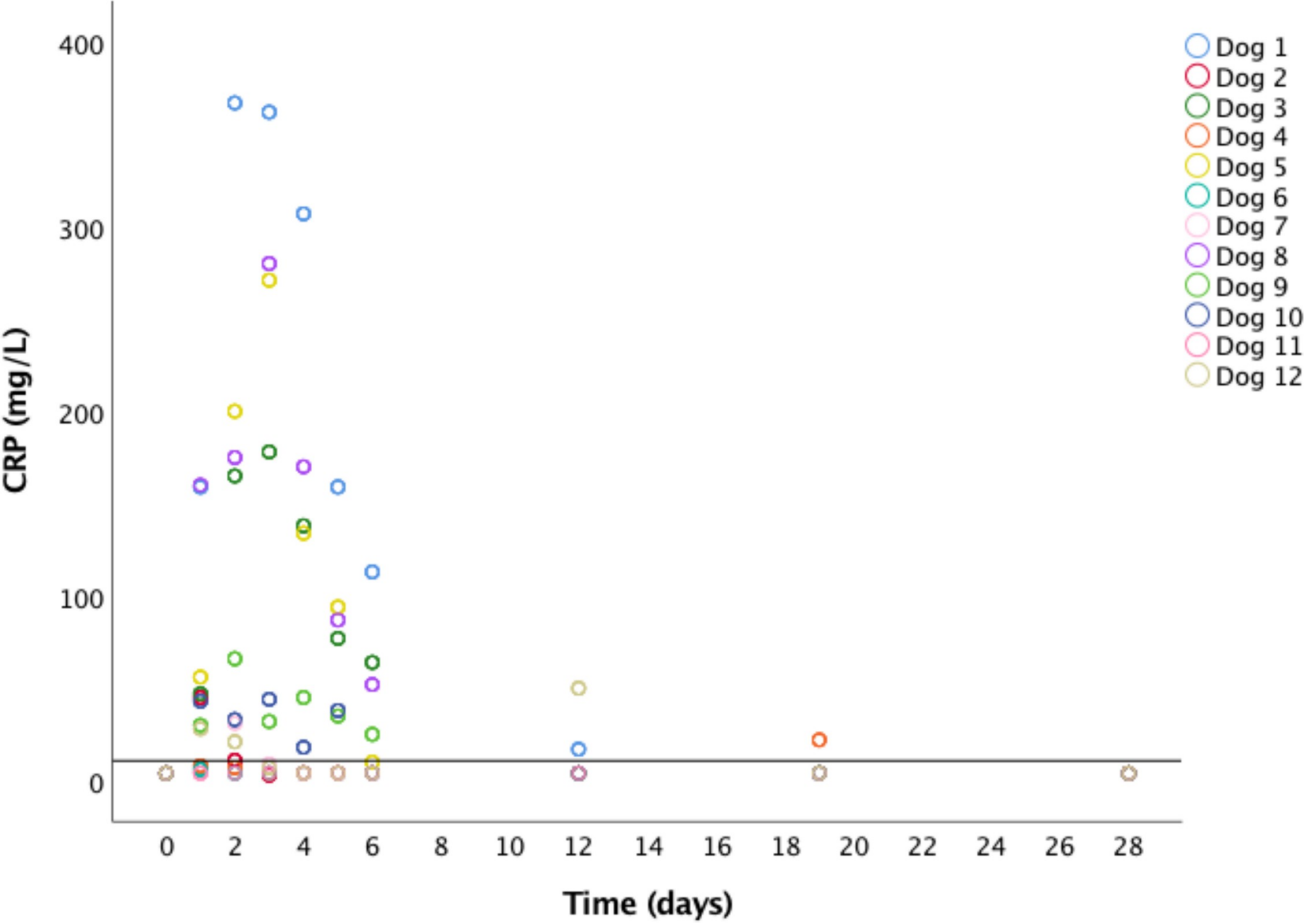
Scatter plot depicting individual C-reactive protein (CRP) values in serum measured from dogs at each post-interventional control at days 1 to 6, 12, 19, and 28. The continuous horizontal red line shows the reference value for CRP (< 10.7 mg/L). No elevations were noticed 4 weeks after intraprostatic steam application.

CRP levels in serum monitored during the first week and 12, 19, and 28 days after steam application are depicted in Fig 3. Six of the seven dogs which showed clinical signs of discomfort within 24 hours after steam application showed elevations of CRP levels in serum as well.

### MRI findings

**Fig 4:**
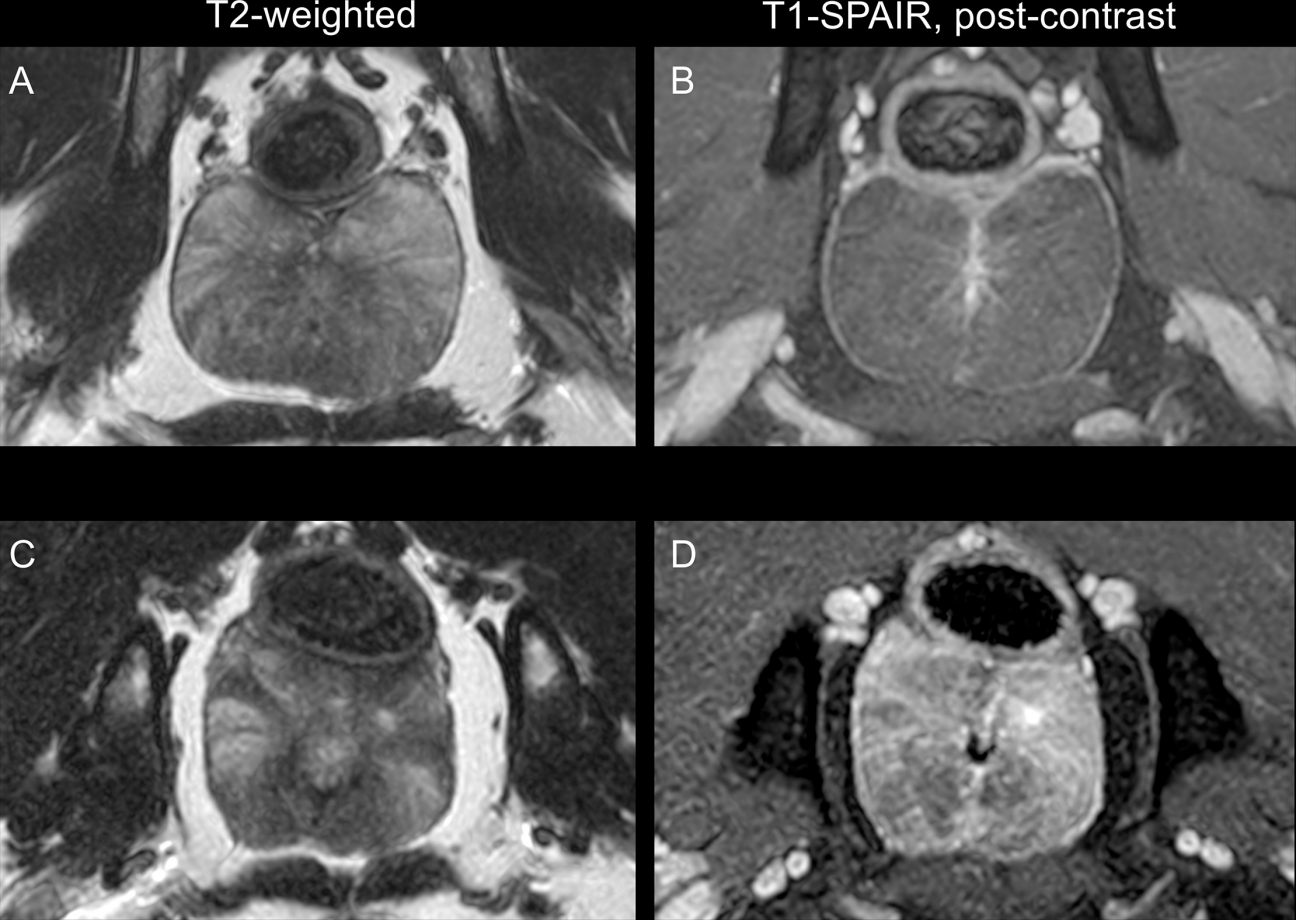
Transverse T2-weighted (T2W) and T1-SPAIR images of the prostate gland of a 6 years-old healthy beagle, before (A and B) and 4 weeks after intraprostatic steam application (C and D). Before intraprostatic steam application, the prostate displays a mild homogeneous hyperintense radiating pattern in T2W and a homogeneous appearance isointense to the muscle with no contrast enhancement in T1-SPAIR sequences, consistent with normal canine MRI appearance. Four weeks post-intervention, moderate, well-defined, multifocal T2 hyperintense intraprostatic lesions are noticed (C); the lesions enhance moderately after contrast agent administration (D).

Before steam application, all prostate glands displayed normal MRI characteristics, being homogenous isointense in T1-weighted and slightly hyperintense to muscle in T2-weighted sequences. Moreover, a homogeneous and mildly increased in T2 signal intensity radiating pattern was conspicuous in dorsal and transverse planes and mild homogeneous-to-none enhancement was present after contrast agent administration, being consistent with normal prostatic parenchyma (Fig 4A and 4B) [27]. The size and contrast enhancement behaviour of the medial iliac lymph nodes was normal at this time point (Table 1).

**Table 1:** Mean values of medial iliac lymph nodes size at each time point and comparison between time points.

	Before treatment Mean ± SD		4 weeks after treatment Mean ± SD		P-value	
	Width (mm)	Height (mm)	Width (mm)	Height (mm)	Width	Height
Group 1 (n = 6)	4.59 ± 0.95	3.91 ± 0.8	5.52 ± 1.59	4.33 ± 1.10	0.89	0.24
Group 2 (n = 6)	6.1 ± 2.11	3.42 ± 0.66	5.83 ± 1.79	3.83 ± 0.95	0.98	0.41

Abbreviation: SD; standard deviation.

Four weeks after intraprostatic steam application, mild T2 hyperintense lesions were found in 9/12 dogs; being focal in two, multifocal in two and diffuse in five dogs. Moderate multifocal increase in T2 signal intensity lesions were evident in 2/12 dogs (Fig 4C).

Regarding signal behaviour in post-contrast SPAIR T1 sequences, mild increased in contrast uptake was evident in 8/12 dogs; being focal in three, multifocal in one and diffuse in four. Moderate multifocal contrast enhancement was present in 2/12 dogs; no cavitary lesions were present in any of the prostate glands of the dogs (Fig 4D). Given the appearance and distribution of the lesions, assessment of differences of severity regarding laterality was not feasible. The medial iliac lymph nodes were not included in the examined field of view in one case. No MRI features consistent with medial iliac lymphadenopathy such as enlargement or increased heterogeneous contrast enhancement were present (Table 1).

### Ultrasonographic follow-up

During the first three post-interventional examinations, lesions within the prostatic parenchyma could not be detected ultrasonographically. Furthermore, on day 6, hyperechogenic radial bands were first noted within the prostatic parenchyma in all dogs. These radial bands became more noticeable by increasing in size and echogenicity until day 23 and persisted faintly until day 27 (S1 Fig) During the last ultrasonographic examination, the prostate was overall generalized hyperechoic compared to the pre-intervention assessment in all dogs. Additionally, alterations of the medial iliac lymph nodes or the colonic wall adjacent to the prostate were not evidenced during ultrasound examination.

### Histopathological evaluation

During biopsy procedure, three samples from each prostatic lobe per dog were taken, for a total number of 72 samples. Prostatic parenchyma or associated lesioned tissue could not be observed in six samples, representing three samples obtained from two prostatic lobes from two different dogs; therefore, a total of 66 samples of prostatic tissue were assessed. Moreover, all prostatic biopsies were assessable, and the quality of the collected tissues was considered to be low in 6/66 (9.1%), moderate in 9/66 (13.6%), good in 26/66 (39.4%), and excellent in 25/66 (37.9%) of the samples.

Presence of a lesion, characterized by necrosis or associated inflammation, was confirmed in 58/66 (87.9%) samples. From these, presence of necrosis and inflammation were detected in 50 and 53 biopsies, respectively. Necrosis was of coagulative nature in all cases, with concurrent fibrinoid component in four of them; whereas inflammation was chronic in all samples.

**Fig 5:**
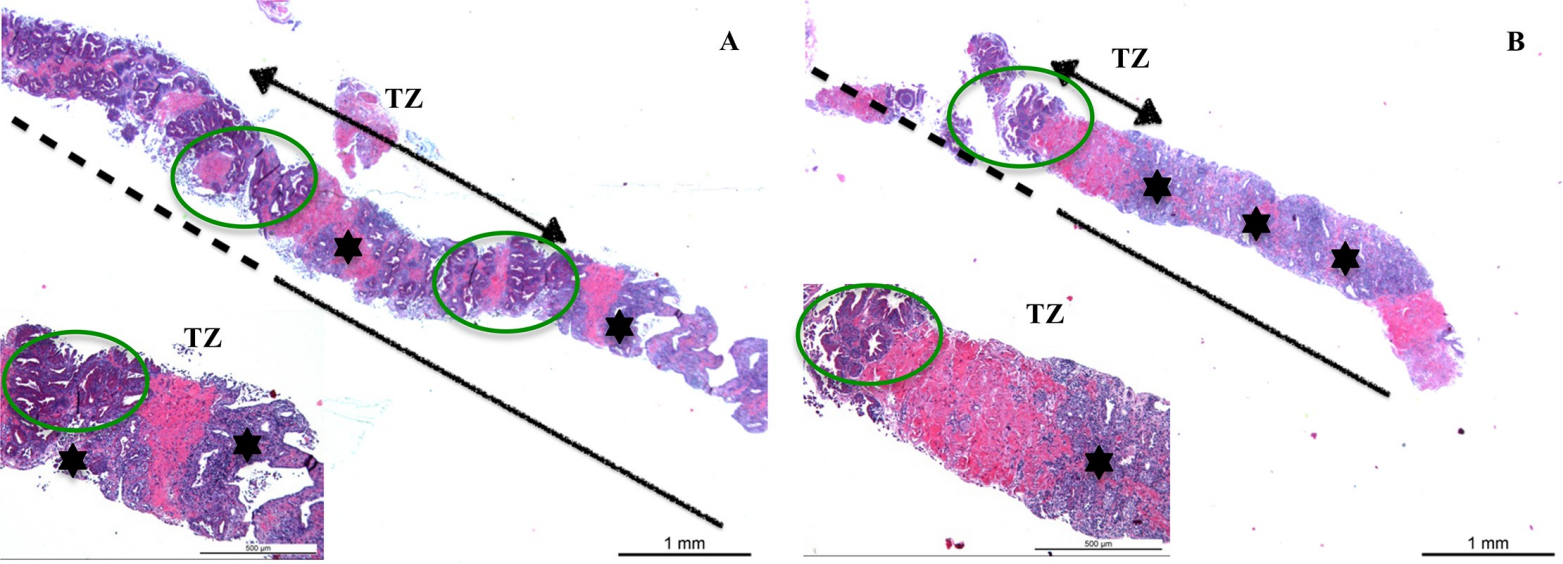
Section of prostate biopsy cores obtained from two healthy beagle dogs 4 weeks after intraprostatic steam application depicting smooth (A) and sharp (B) transition zones (TZ). TZ are represented by a black arrow between treated (continuous black line) and untreated areas (discontinued black line). Areas of inflammation and necrosis (black asterisk) intermingled within normal prostatic tissue (green oval) are noticed in A, whereas a clear interruption of between normal prostatic tissue and areas of inflammation and necrosis are depicted in B. *HE staining*, *magnification 1*.*25x10*.

Transition zone (TZ) defect could be identified in 18 biopsies and was classified as sharp in 7 and smooth in 11 samples (Fig 5). Furthermore, presence of fibrosis within the prostatic parenchyma was detected in 55/66 (83.3%) samples. Fragments of rectal mucosa were found in 32/66 (48.5%) prostatic biopsies.

**Fig 6:**
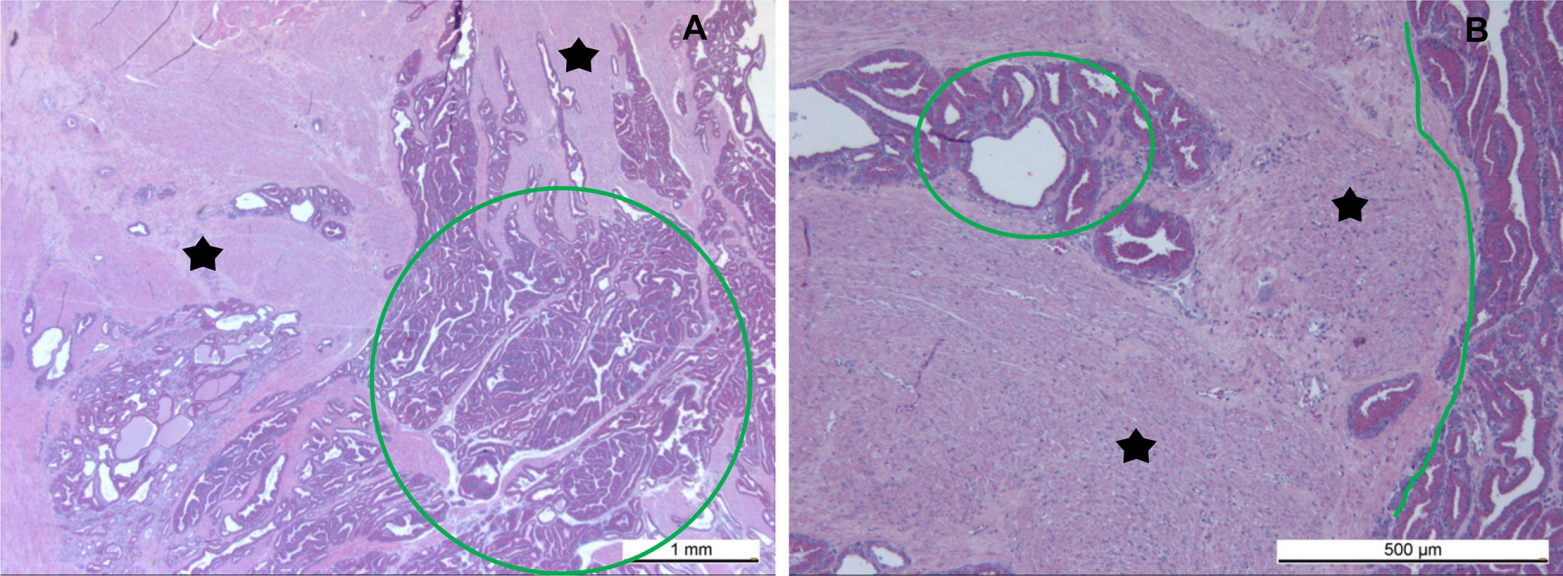
Sections of the prostate (from the whole organ) 11 months after the intraprostatic steam application, (A) an overview of the irregular but sharp transition zone from the steam treated area (black asterisk) to the normal glandular tissue (green oval), (B) higher magnification of the steam-treated glandular tissue, which was replaced by broad, collagen-rich fibrous tissue without any signs of inflammation or necrosis (scar). *HE staining*, *magnification 1*.*25x10 (A)*, *5x10 (B)*.

The histopathological evaluation of the single complete prostate gland obtained eleven months after intraprostatic steam application revealed the presence of an irregular in contour, but sharply marginated TZ. The treated prostatic parenchyma was replaced by broad, collagen-rich fibrous tissue without any signs of inflammation or necrosis (Fig 6).

## Discussion

Feasibility of transrectal, MRI guided thermal prostate ablation using water vapour was proven in 12 healthy beagle dogs. Clinically, the dogs showed mild levels of discomfort according to the CMPS-SF pain score and no additional analgesic intervention had to be considered. As expected, CRP levels increased in the acute phase after intraprostatic steam was applied. Systemic inflammatory response, indicated by levels of CRP in serum, may play an important role in the classification and prognosis of PCa in humans before undergoing surgical treatment or radiotherapy [28–30]. Understanding the temporal behaviour of CRP in serum after focal prostatic ablation is therefore necessary for future research in prostatic pathologies utilizing the canine model.

The fact that lesions induced by steam therapy were more conspicuous in MRI than conventional ultrasound follow-up examinations is consistent with previous reports in beagle dogs [15]. Lesions were identified as mild and diffusely distributed in most of the cases in MRI evaluations (9/12 and 5/12, respectively); however, multifocal distribution of lesions within the prostate were also present in some cases (2/12). A possible explanation of the variable distribution of the lesions could be the limited number of pulses applied, which were not sufficient to produce a cavitary lesion. Additionally, this feature limited a direct comparison of the lesion size between the different number of pulses applied and the predictability of tissue sampling.

As expected, neither an increased signal intensity within the periprostatic tissues nor medial iliac lymphadenomegaly were noticed in the follow-up MRI examination. These findings are consistent with the clinical examination and pain score assessment at that time point.

Transrectal MRI-guided biopsy procedures have demonstrated to have higher accuracy in obtaining diagnostic biopsies of lesions detected in MRI in humans in comparison to ultrasound-guided methods [31, 32]. Although post treatment MRI findings were variable and only partially consistent among dogs, histopathological confirmation of prostatic parenchymal lesion caused by thermal ablation was achieved in 80.6% (58/72) of the samples obtained in the present study. This finding reflects similar diagnostic accuracy of this technique obtaining biopsies of lesions detected in MRI reported in humans with PCa [3]. Moreover, although available for a single case, the results from the long-term histopathologic examination suggest a stable effect of the coagulative necrosis in the prostatic tissue, with predominant presence of fibrosis and absence of active or chronic-active inflammatory changes.

The multifocal nature of the lesions and presence of smooth transition zones within the obtained biopsies could be explained due to interstitial distribution of high-temperature condensed water vapour within the tissue or convective thermal distribution, which is characteristic of steam-induced ablation [33].

Prostatic thermal ablation using water vapour is a promising technique that has shown an improvement of clinical signs related with BPH in humans [34]; however, urinary incontinence or retention have been reported during the first week post intervention, probably due to transurethral approach [35]. Conversely to transurethral approaches, transrectal MRI-targeted approach allows focal treatment of peripheral lesions without compromising the integrity of the urethra and adjacent normal appearing prostatic tissues. Additionally, this approach allows multiplanar and real time MRI verification of the position of the needle within the tissue, diminishing misregistration issues that commonly affect fusion imaging [3].

Since the implementation of the prostatic-specific antigen (PSA) test as a standard biomarker for PCa, overtreatment of affected men by means of radical prostatectomy has increased, which in many cases can be clinically irrelevant [5]. As PSA cannot reliably distinguish between PCa and BPH, the overtreatment scenario applies for the latter condition as well [36]. Furthermore, reported middle-term occurrence rates of urinary incontinence and erectile dysfunction after radical prostatectomy up to 29% and 59%, respectively [37]. Focal intraprostatic therapies and a narrowed selection of the population to be treated, namely recruitment of men with intermediate or high-risk disease, represent an alternative to overcome these issues [38].

As demonstrated in the present study, 10 and 15 pulses were sufficient to reliably produce coagulative necrosis within the prostate gland parenchyma. However, a well-defined cavitary necrotic lesion was not produced with this number of pulses and therefore no volumetric analysis of the treated area could be performed, which could be considered a limitation. From an ethical point of view, a minimal effective steam pulse number was determined in *ex-vivo* experiments and applied to the experimental population, as the study was designed for middle term follow-up assessments.

In addition to anatomic similarities, BPH and PCa occur spontaneously in dogs [23, 24, 39]. This study sets a baseline for future research regarding intraprostatic steam application in a population of dogs affected by prostatic pathologies, as a target, naturally occurring, translational population for diseases such as BPH and PCa. Enhancing the understanding of the role of novel focal therapeutic approaches for prostatic diseases in a large animal translational model benefits both, private-owned dogs and men affected by prostatic diseases.

In conclusion, MRI-targeted intraprostatic steam application is a feasible and promising technique and displays minimal side effects in a large animal translational model for prostatic diseases in humans.

## Supporting information

S1 Fig Ultrasound images in the longitudinal axis of the prostate gland of a 3 years-old healthy beagle, before (A), 13 (B), and 27 days (C) after intraprostatic steam application. Intraparenchymal hyperechoic radiating bands are noticed in B and C (white arrowheads).
(TIFF)

## Abbreviations

BPH: benign prostatic hyperplasia
CBC: complete blood count
CMPS-SF: short form of the Glasgow composite measure pain scale
CRP: C-reactive protein
MRI: magnetic resonance imaging
PCa: prostatic cancer
PSA: prostatic specific antigen
SPAIR: spectral attenuated inversion recovery
T1W: T1-weighted
T2W: T2-weighted
