# Supplementary figures and images for: MRI-guided, transrectal, intraprostatic steam application as potential focal therapeutic modality for prostatic diseases in a large animal translational model: A feasibility follow-up study
Source: PLoS One. 2019 Dec 23;14(12):e0226764. doi: 10.1371/journal.pone.0226764 (PMC6927626; doi:10.1371/journal.pone.0226764)

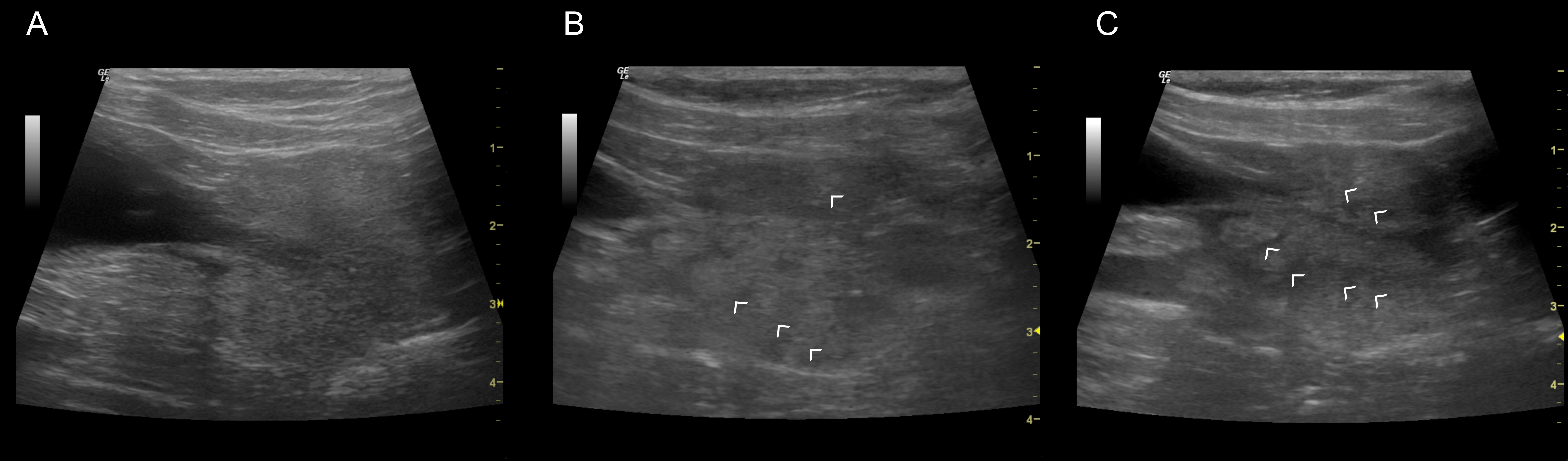

Supplement: S1 Fig — Ultrasound images in the longitudinal axis of the prostate gland of a 3 years-old healthy beagle, before (A), 13 (B), and 27 days (C) after intraprostatic steam application. Intraparenchymal hyperechoic radiating bands are noticed in B and C (white arrowheads). (TIFF) [file pone.0226764.s001.tiff]
